# Profile of Hepatic Encephalopathy in Children with Cirrhosis and Response to Lactulose

**DOI:** 10.4103/1319-3767.77246

**Published:** 2011

**Authors:** Praveen Sharma, Barjesh C. Sharma

**Affiliations:** Department of Gastroenterology, G. B. Pant Hospital, New Delhi, India

**Keywords:** Children, cirrhosis, hepatic encephalopathy, lactulose

## Abstract

**Background/Aim:**

Hepatic encephalopathy (HE) is associated with a poor prognosis. There is paucity of data on the treatment of HE with lactulose in children with cirrhosis.

**Patients and Methods:**

Retrospective analysis of consecutive cirrhotic patients (<18 years) with HE was done. HE was defined according to West-Haven criteria. Response was defined as complete if patients recovered completely from HE, partial response was defined as improvement of encephalopathy by one or more grades from admission but not complete recovery, and defined as non response if patient did not show any improvement or deteriorated further even after 10 days of lactulose therapy.

**Results:**

A total of 300 patients were admitted with cirrhosis and HE (278 adults and 22 children). Of 22 patients, 16 (73%) patients had complete response to lactulose and six (27%) patients did not [three (13.5%) patients worsened (non response) and three (13.5%) did not recover fully even after 10 days of treatment (partial response)]. Comparing baseline characteristics of patients who had complete response (*n*=16) versus partial (*n*=3) and non response (*n*=3), there was significant difference in mean arterial pressure (78.1±10.7 *vs* 62.6±5.0 mmHg, *P*=0.003), serum sodium (131.3±3.2 *vs* 126.5±5.2, *P*=0.01) and serum creatinine (0.78±0.3 *vs* 1.1±0.3 mg/dl, *P*=0.02). We did not find any difference in baseline characteristics of these patients regarding CTP score (9.6±1.2 *vs* 10.6±1.2), MELD score (17.6±2.9 *vs* 17.1±3.4), severity of HE (2.5±0.6 *vs* 2.6±0.5) and etiology of precipitating factors (*P*=0.78).

**Conclusions:**

Lactulose therapy causes complete recovery from hepatic encephalopathy in 73% of pediatrics patients with cirrhosis.

Hepatic encephalopathy (HE) refers to neuropsychiatric abnormalities that result from hepatic dysfunction. It is one of the principal manifestations of chronic liver disease.[1] West Haven criteria is often used for the assessment of HE which is characterized by [grade 1 (mild lack of awareness and shortened attention span), grade 2 (lethargic, disoriented, inappropriate behavior, obvious asterixis and slurred speech), grade 3 (somnolent but arousable, gross disorientation, bizarre behavior, muscular rigidity, clonus and hyperreflexia) and grade 4 (coma and decerebrate posturing).[1] Numerous factors have been shown to precipitate HE including infections, sedatives, gastrointestinal bleeding, dietary protein excess, diuretics, and electrolyte imbalance.[2] The primary therapeutic diction is the identification and treatment of the precipitating factors. The therapeutic approach to HE is aimed at decreasing ammoniagenic substrates and at inhibiting ammonia generation, as well as at reducing its intestinal absorption and facilitating its elimination. This includes a reduction of proteins in the diet, the administration of non-absorbable disaccharides, and/or the administration of non-absorbable antibiotics.[3–5] Non-absorbable disaccharides are often used as first-line pharmacotherapy. Disaccharides remain undigested until they reach the colon, where they function to inhibit bacterial ammonia production and trap ammonia as non-diffusible ammonium in the intestinal lumen.[6–10] Some studies have shown that lactulose/lactitol is effective in 60-80% cases in the treatment of acute portosystemic HE.[6,9] A small meta analysis determined that lactulose and lactitol were equally effective in the treatment of HE.[10] A meta-analysis of 22 randomized trials highlighted the lack of data supporting the efficacy of nonabsorbable disaccharides.[11] Oral poorly absorbable antibiotics are an alternative for pharmacological treatment of HE but they have their own limitations like development of ototoxicity and nephrotoxicity with neomycin, bad oral taste and neuropathy with long-term metronidazole.[8,12,13] Lactulose is still preferred as a first line therapy for the treatment of HE in adults as well as children by most physicians. However, there is hardly any data on the treatment of HE with lactulose in chronic liver disease in children. We describe our experience with lactulose in the treatment of HE in cirrhosis in pediatrics patients.

## PATIENTS AND METHODS

### Patient population

Retrospective analysis of patients (<18 years) who were hospitalized with HE due to liver cirrhosis during a three-year period between January, 2006 and January, 2009 at G. B. Pant Hospital, New Delhi was done. Cirrhosis was diagnosed on a clinical basis involving laboratory tests, endoscopic evidence, sonographic findings and liver histology if available. Patients were excluded from the study if they had nonhepatic metabolic encephalopathies, severe co morbid conditions (like severe associated lung or cardiac disease) or hepatic encephalopathy due to acute liver failure. We collected demographic information, CTP class, MELD score, blood urea and serum creatinine, etiology of cirrhosis, ascitic fluid data including cell count and protein of the paracentesis performed at the time of admission, (bacteriologic confirmation of spontaneous bacterial peritonitis if available), abdominal ultrasound with Doppler/CT of abdomen for the presence of spontaneous shunts, presence of hepatocellular carcinoma, blood and urine culture and chest X-ray of each patient. Ascites was graded according to 0 (none), 1 (mild), and 2 (moderate to severe).

### Management of hepatic encephalopathy and other complications of portal hypertension

All patients were admitted in intensive liver unit with monitoring of the vital functions, electrolytes and acid-base status. Stopping of gastrointestinal bleedings, preventing benzodiazepine, sedatives, and specific diuretics overdosage or a protein-overload was done. All the patients were treated with lactulose in dosage so that patients had two-three semiformed stools/day. Patients who had spontaneous bacterial peritonitis (SBP) were treated with cefotaxime for five days and albumin as per the standard protocol and later were put on secondary prophylaxis of SBP with norfloxacin. Patients admitted with variceal bleed were also given ceftriaxone for five days. Similarly admissions for other infective causes were treated with antibiotics according to sensitivity of organisms.

### Assessment and recovery of hepatic encephalopathy

HE was defined according to West-Haven criteria.[1] Response to lactulose was assessed clinically by two consultants with more than ten years of experience in hepatology. Response was defined as complete if patients recovered completely, partial response was defined as improvement of encephalopathy by one or more grades from admission but not complete recovery, and defined as nonresponse if patient did not show any improvement or progressed to deeper grade of HE as per West-Haven criteria after 10 days of lactulose therapy.[14] Patients were followed up for one month since the day of admission.

### Statistical analysis and data management

Data were expressed as mean ± S.D. For a comparison of categorical variables, chi-square and Fisher’s exact tests were used, and for continuous variables, Mann-Whitney test for unpaired data and Wilcoxon rank sum test for paired data were used as appropriate. Intention to treat (ITT) analysis was also done. Significance level of 0.05 was used in all analyses. The statistical analysis was done using SPSS Version 10.0 software (SPSS, Chicago, IL,)

## RESULTS

From January 2006 to January 2009, a total of 300 patients were admitted with cirrhosis and HE (278 adults and 22 children). Etiology of cirrhosis in children was due to autoimmune (*n*=8), hepatitis B (*n*=8) and cryptogenic (*n*=4) and Wilson’s disease in two patients. The clinical and demographic characteristics of the patients enrolled are shown in Table 1.

**Table 1:** Demographic, clinical and biochemical characteristics of 22 study patients

Age (years)	13.5±2.5
Sex (M:F)	8:14
Total bilirubin mg/dl (median, range)	4.7 (0.9-31.0)
AST (U/L) (median, range)	87 (39-350)
ALT (U/L) (median, range)	76 (32-450)
Blood urea (mg/dl)	28.0±12.6
Serum creatinine (mg/dl)	0.9± 0.3
Serum sodium (mmol/l)	130.8±4.2
Ascites (none/mild/moderate-severe)	3:8:11
Hemoglobin (g/dl)	9.7±1.9
TLC (per cubic mm)	8092±2843
Hepatorenal syndrome	2 (9%)
Large spontaneous shunts	3 (14%)
Precipitant factors of HE	
Gastrointestinal bleeding	5 (23%)
Spontaneous bacterial peritonitis and	
other infections*	6 (27%)
Unknown	7 (32%)
Constipation	3 (14%)
Protein overload	1 (4%)
MELD score	17.5±3.0
CTP score	9.9±1.3
MAP mmHg	84.5±9.6
Frusemide mg/d	46.3±15.5
Sprinolactone mg/d	88.6±46.1
HE severity (grade1:2:3:4)	0:10:11:1

* Two patients had spontaneous bacterial peritonitis and chest infection. MELD: Model for end stage liver disease; CTP: Child-Turcotte-Pugh; HE: Hepatic encephalopathy; MAP: Mean arterial pressure; AST: aspartate aminotransferase; ALT: alanine aminotransferase; TLC: total leukocyte count

### Response to lactulose

Of 22 patients, 16 (73%) patients had complete response to lactulose and six (27%) patients did not [three (13.5%) patients worsened (non responder) and three (13.5%) did not recover fully even after 10 days of treatment (partial response)]. Comparing baseline characteristics of patients who had complete response (*n*=16) versus partial (*n*=3) and non response (*n*=3) there was significant difference in mean arterial pressure (78.1±10.7 *vs* 62.6±5.0 mmHg, *P*=0.003),serum sodium (131.3±3.2 *vs* 126.5±5.2, *P*=0.01) and serum creatinine (0.78±0.3 *vs* 1.1±0.3 mg/dl, *P*=0.02).We did not find any difference in baseline CTP score (9.6±1.2 *vs* 10.6±1.2), MELD score (17.6±2.9 *vs* 17.1±3.4), severity of HE (2.5±0.6 *vs* 2.6±0.5) [Table 2].

**Table 2:** Comparison of baseline characteristics between patients with complete response versus those with nonresponse and partial response

	Complete response *n*=16	Nonresponse and partial response *n*=6	*P* value
Age (years)	12.8±2.6	15.1±1.2	0.06
TLC (mm^3^)	7545.6±2415.7	9550.0±3597.6	0.14
Mean arterial pressure (mmHg)	78.1±10.7	62.6±5.0	0.003
CTP score	9.6±1.2	10.6±1.2	0.11
MELD score	17.6±2.9	17.1±3.4	0.76
Na (mmol/l)	131.3±3.2	126.5±5.2	0.01
ALT (IU/l)	96.3±66.7	199.5±194.5	0.07
AST (IU/l)	111.4±73.2	134.3±111.8	0.57
Urea (mg/dl)	28.1±10.5	27.5±18.3	0.92
Creatinine (mg/dl)	0.78±0.3	1.1±0.3	0.02
Frusemide (mg/day)	50.0±16.6	36.6±8.1	0.07
Spironolactone (mg/day)	96.8±49.8	66.6±25.8	0.17
HE grade	2.5±0.6	2.6±0.5	0.72
Large spontaneous shunts	2	1	0.80
Precipitating factor			
Infections	5	1	0.78
Bleed	4	1	
Unknown	4	3	
Constipation	2	1	
Protein overload	1	0	

MELD: Model for end stage liver disease; CTP: Child-Turcotte-Pugh; HE: Hepatic encephalopathy; MAP: Mean arterial pressure; AST: aspartate aminotransferase; ALT: alanine aminotransferase; TLC: total leukocyte count

Non responder patients (*n*=3) died during follow up [day 4, day 7 and 8) of progressive liver failure while partial responder patients (*n*=3) recovered from HE on prolonged therapy and were discharged (day 16, 21 and 22).

### Etiology of precipitating factor for hepatic encephalopathy and response to lactulose

Of 22 patients with HE, the precipitating factors were {upper gastrointestinal bleed, *n*=5 (23%), infections, *n*=6 (27%)[(pneumonia, *n*=2, pneumonia with SBP, *n*=2), (urinary tract infection, *n*=2)], constipation, *n*=3 (14%), protein overload, *n*=1 (4%) and unknown causes, *n*=7 (32%)}. Of 16 patients who had complete response, infections were seen in five patients compared to one in non responders; however, there was no significant difference between the precipitating factor between the two groups (*P*=0.78) [Table 2].

## DISCUSSION

In this study, complete recovery from HE was seen in 73% of patients with cirrhosis within 10 days of lactulose therapy.

The clinical features of HE are well defined in adults, but not for children. According to one classification HE in children is defined as: stage I, inconsolable crying; stage II, inconsolable crying and inattention to task; stage III, somnolence, stupor, combativeness; stage IV, comatose, arousal with painful stimuli (IVa) or no response (IVb).[15] However, in our cohort we used West-Haven criteria for assessment of HE as it is still widely used for HE. Serum ammonia remains the surrogate marker for encephalopathy, but altered ammonia metabolism is only a component of the complex nature of this clinical entity.[16 17] A nonabsorbable antibiotic such as neomycin is not commonly used in children, as long-term use is associated with nephro- and ototoxicity.[8,13]

Lactulose is often used as a first line therapy for the treatment of HE with cirrhosis in adults as well as children. Studies have shown that lactulose/lactitol is effective in 60-80% in the treatment of acute portosystemic HE in adults.[6,8,9] Similarly, in our study, complete recovery from HE was seen in 73% and partial recovery in 14% of children. Thus, we found lactulose to be equally effective in the management of HE in children.

Hyponatremia is known to be associated with morbidity and early mortality.[18–20]

A recent hypothesis proposes the role of low-grade cerebral edema in the pathogenesis of HE and MHE.[21] Guevera *et al*.[22] found that, in patients with cirrhosis, the existence of hyponatremia is a major risk factor of the development of overt HE. In this study, we found baseline sodium was significantly lower in nonresponders compared to responders (126.5±5.2 *vs* 131.3±3.2 mmol/l, *P*=0.01).

Hemodynamic dysregulation is more pronounced with increasing severity of the liver disease.[23] In this study, patients with non response to lactulose had significantly lower mean arterial pressure than patients who responded to lactulose (62.6±5.0 *vs* 78.1±10.7 mmHg, *P*=0.003). We also found patients with nonresponse to lactulose had higher baseline serum creatinine as compared to patients who responded to lactulose (1.1±0.3 *vs* 0.78±0.3 mg/dl, *P*=0.02). Since both, hepatorenal syndrome and hyponatremia are independent predictors of mortality in cirrhotic patients, it may have created a type II error. We did not find any difference in grades of HE between responders versus nonresponders to lactulose. The grade of encephalopathy did not predict the mortality in both acute and chronic liver disease. There are other features of liver cell failure, such as bilirubin, prothrombin time, and underlying cause of liver disease, that interact and finally determine the outcome. Also, in some cases, encephalopathy is precipitated by reversible factors such as constipation, diuretic use, therapeutic paracentesis, and electrolyte disturbances that may be reversible by treatment and hence the grade alone is not an indicator of the severity of liver disease.

Lactulose is often used as first line therapy both in adults and in children who developed HE due to cirrhosis.[24] Though there is some evidence for its use in adults, its use in pediatrics patients has not been well documented. This study highlights the role of lactulose in a pediatric cirrhotic population and to the best of our knowledge, is the largest study of its kind. A limitation of this study is that we did not analyze arterial ammonia or gut flora changes in these patients for definitive evidence of the role of lactulose in these patients. However, these tests are not done routinely in the management of these patients. To conclude, lactulose is effective in the management of hepatic encephalopathy in children with cirrhosis.

## References

[1] Ferenci P, Lockwood A, Mullen K, Tarter R, Weissenborn K, Blei AT (2002). Hepatic encephalopathy--definition, nomenclature, diagnosis, and quantification: final report of the working party at the 11th World Congresses of Gastroenterology, Vienna, 1998. Hepatology.

[2] Riordan SM, Williams R (1997). Treatment of hepatic encephalopathy. N Engl J Med.

[3] Jalan R, Hayes PC (1997). Hepatic encephalopathy and ascites. Lancet.

[4] Blei AT, Córdoba J (2001). Practice Parameters Committee of the American College of Gastroenterology. Hepatic encephalopathy. Am J Gastroenterol.

[5] Uribe M, Berthier JM, Lewis H, Mata JM, Sierra JG, García-Ramos G (1981). Lactose enemas plus placebo tablets versus neomycin tablets plus starch enemas in acute portal systemic encephalopathy.A doubleblind randomized controlled study. Gastroenterology.

[6] Blanc P, Daures JP, Rouillon JM, Peray P, Pierrugues R, Larrey D (1992). Lactitol or lactulose in the treatment of chronic hepatic encephalopathy: results of a meta-analysis. Hepatology.

[7] Cordoba J, Blei AT (1997). Treatment of hepatic encephalopathy. Am J Gastroenterol.

[8] Morgan MY, Hawley KE (1987). Lactitol vs.lactulose in the treatment of acute hepatic encephalopathy in cirrhotic patients: a double-blind, randomized trial. Hepatology.

[9] Paik HY, Lee KS, Han KH, Song KH, Kim MH, Moon BS (2005). Comparison of rifaximin and lactulose for the treatment of hepatic encephalopathy: a prospective randomized study. Yonsei Med J.

[10] Camma C, Fiorello F, Tine F, Marchesini G, Fabbri A, Pagliaro L (1993). Lactitol in treatment of chronic hepatic encephalopathy.A meta-analysis. Dig Dis Sci.

[11] Als-Nielsen B, Gluud LL, Gluud C (2004). Nonabsorbable disaccharides for hepatic encephalopathy: systematic review of randomised trials. BMJ.

[12] Loft S, Sonne J, Døssing M, Andreasen PB (1987). Metronidazole pharmacokinetics in patients with hepatic encephalopathy. Scand J Gastroenterol.

[13] Morgan MH, Read AE, Speller DC (1982). Treatment of hepatic encephalopathy with metronidazole. Gut.

[14] Mas A, Juan R, Sunyer L, Rodrigo L, Planas R, Vargas V (2003). Comparison of rifaximin and lactitol in the treatment of acute hepatic encephalopathy: results of a randomized, double-blind, double dummy, controlled clinical trial. J Hepatol.

[15] Squires RH (2001). End-stage liver disease in children. Curr Treat Options Gastroenterol.

[16] Butterworth RF (1996). The neurobiology of hepatic encephalopathy. Semin Liver Dis.

[17] Norenberg MD (1996). Astrocytic-ammonia interactions in hepatic encephalopathy. Semin Liver Dis.

[18] Heuman DM, Abou-assi SG, Habib A, Williams LM, Stravitz RT, Sanyal AJ (2004). Persistent ascites and low serum sodium identify patients with cirrhosis and low MELD scores who are at risk for early death. Hepatology.

[19] Biggins SW, Rodriguez HJ, Bacchetti P, Bass NM, Roberts JP, Terrault NA (2005). Serum sodium predicts mortality in patients listed for liver transplantation. Hepatology.

[20] Borroni G, Maggi A, Sangiovanni A, Cazzaniga M, Salerno F (2000). Clinical relevance of hyponatraemia for the hospital outcome of cirrhotic patients. Digest Liver Dis.

[21] Haussinger D (2006). Low grade cerebral edema and the pathogenesis of hepatic encephalopathy in cirrhosis. Hepatology.

[22] Guevara M, Baccaro ME, Torre A, Gómez-Ansón B, Ríos J, Torres F (2009). Hyponatremia is a risk factor of hepatic encephalopathy in patients with cirrhosis: a prospective study with time-dependent analysis. Am J Gastroenterol.

[23] Loyke HF (1955). The relationship of cirrhosis of the liver to hypertension: a study of 504 cases of cirrhosis of the liver. Am J Med Sci.

[24] Ullrich D, Birchner J, Conn HO, Birchner J (1988). HLactulose in pediatrics. Hepatic Encephalopathy: Management with Lactulose and Related Carbohydrates.

